# Effect of preoperative vitamin D deficiency on postoperative hypocalcemia after thyroid surgery

**DOI:** 10.1186/1756-6614-7-8

**Published:** 2014-12-15

**Authors:** Mayank Tripathi, Rajender Kumar Karwasra, Sanjeev Parshad

**Affiliations:** Department of Surgical Oncology, Pt.B.D.Sharma PGIMS and UHS, Rohtak, Haryana, 124001 India; Department of Surgery, Pt.B.D.Sharma, PGIMS and UHS, Rohtak, Haryana, 124001 India

## Abstract

**Background:**

Transient post thyroidectomy hypocalcemia occurs in up to 30% of patients. We evaluated the effect of vitamin D deficiency on post thyroidectomy hypocalcaemia.

**Methods:**

This is a prospective study which was conducted from November 2010 to January 2013 and a total of 35 patients were included and data was analyzed regarding the relation between preoperative vitamin D3 levels and occurrence of post- thyroidectomy hypocalcemia. Patients were divided into two groups dependent upon the preoperative serum vitamin D level: group 1 with vitamin D levels <20 ng/ml and group 2 with serum vitamin D levels ≥20 ng/ml. Hypocalcemia was defined as a postoperative calcium level <8.5 mg/dl.

**Results:**

There was a difference in postoperative hypocalcemia between the two vitamin D groups. In patients with serum vitamin D ≤20 ng/ml mean pre-operative and post-operative serum calcium levels were 9.3 ± 0.5 and 8.4 ± 0.58 g dl (p < .001) whereas in patients with serum vitamin D levels >20 ng/ml mean pre-operative and post-operative serum calcium were 9.52 ± 0.64 and 8.9 ± 0.5 (p = ns).

**Conclusions:**

Pre-operative serum vitamin D levels have got positive correlation with serum calcium levels in early post-operative period. Patients with serum vitamin D levels <20 ng/ml are highly likely to develop early post-operative hypocalcaemia and the difference between pre-operative and post-operative serum calcium levels in vitamin D deficient patients was significant (p < 0.001).

## Introduction

Hypocalcemia is a well-known complication and concern following thyroid surgery. Although in most cases it is only temporary, post-thyroidectomy hypocalcemia can lead to an increased cost by prolonging the length of stay and increasing the need for expensive medications, frequent biochemical tests and multiple outpatient visits [1]. The incidence of transient hypocalcemia has been estimated to occur between 3% to 30% of cases even after preservation of one or more parathyroids [2]. Permanent hypocalcaemia, although much less frequent, still occurs, with an incidence of around 2-4% reported in the literature [3]. Only a small percentage of patients with biochemical hypocalcemia develop symptoms, which most commonly manifest as mild perioral or distal acral paresthesias. Less frequently, patients may develop more severe symptoms such as carpopedal spasm, tetany, laryngospasm or in rare cases, cardiac arrhythmias [2]. Although a common occurrence, causes and mechanisms of post thyroidectomy hypocalcemia are unclear. It was first attributed to injury, devascularization, or inadvertent removal of the parathyroid glands. However, the high frequency of post thyroidectomy hypocalcemia even after lobectomy led some authors to challenge the traditional explanation, as it seemed unlikely that the parathyroid glands could be injured or damaged in almost all thyroidectomies [4].

Many factors are postulated to increase the risk of hypocalcemia, including old age Graves’ disease, surgical techniques, concurrent neck dissection, large surgical volumes, and the surgeon’s experience, which have all been shown to increase the risk of hypocalcemia [4–6]. Recently few studies have suggested the role of 25-hydroxycholecalciferol (vitamin D3) deficiency in the occurrence of hypocalcaemia after thyroidectomy [4]. Intuitively, prescribing vitamin D and calcium prophylactically after thyroidectomy has shown to reduce the incidence of hypocalcaemia [7, 8]. It has also been suggested that the combination of calcitriol and hydrochlorothiazide after thyroidectomy may reduce the risk of hypocalcemia [8].The health implications of vitamin D deficiency have become a key area of research. Vitamin D deficiency has been associated with bone disease, malignancy, lipid metabolism defects, diabetes, and heart disease. Reference ranges for normal vitamin D levels and the levels at which pathological correlation can be made are not well established and vary from institution to institution. Demonstrating a correlation between preoperative vitamin D levels and occurrence of post thyroidectomy may reveal an easily correctable factor.

Following ethical approval this prospective study was conducted at a tertiary care hospital on patients who presented with goiter. Patients with evidence of hyperthyroidism, concomitant parathyroid disease, metabolic bone disease and patients with previous thyroid or neck surgery were excluded from the study.

### Measurements

Pre-operative thyroid function tests were performed in all the patients and only euthyroid patients were taken up for surgery. Total Serum calcium, PTH, 25-OHD, and albumin levels were determined the day before surgery. Serum calcium (ca^2+^) and albumin levels were determined by an auto analyzer. Serum 25-OHD and PTH (parathormone) levels were determined by ELISA using commercially available kits. Thyroidectomies were performed by consultant surgeons, during surgery recurrent laryngeal nerves were preserved and an attempt was made to identify all parathyroid glands and to preserve their blood supply by meticulous dissection. In case ischemic changes were noted parathyroids were re-implanted in the ipsilateral sternocleidomastoid muscle. Serum ca^2+^ and PTH levels were measured 12 hours postoperatively, and measurements of serum ca^2+^ levels were repeated 24 hours postoperatively. Repeated measurement of serum ca^2+^ was performed at 1 week, 2 week, 1 month, 2 months and 3 months in post-operative period. Albumin corrected calcium was calculated by adding 1 mg/dl to the serum calcium level for every 1 gm/dl by which serum albumin level is below 4 gm/dl. Any sign of hypocalcemia in the form of facial paresthesia, positive Chvostek or Trousseau signs, and muscular spasm were noted in the pre-operative as well as in the immediate post-operative period and on follow-up. Reference ranges of biochemical parameters are as follows: 8.5 to 10.5 mg/dL for serum ca^2+^; 3.5 to 5.0 g/dL for serum albumin, 20-100 ng/mL for serum 25-OHD (vitamin D) and 10 to 65 pg/mL for serum PTH. Hypocalcemia was defined as serum ca^2+^ levels less than normal reference range in our institute i.e. <8.5 mg/dl. Transient post-operative hypocalcemia was defined as hypocalcemia occurring in first 48 hrs after the surgery and patient was considered as having permanent hypocalcemia if clinical or biochemical evidence of hypocalcaemia were present at 3 month follow-up. Vitamin deficiency was defined as serum vitamin D levels <20 ng/ml. All of the patients who developed asymptomatic hypocalcemia were treated with oral calcium carbonate (3-6 g/d). Symptomatic hypocalcemia was treated with parenteral calcium gluconate and an oral calcitriol supplementation of 1.0 to 1.5 μg/d. Patients were divided in two groups based on their serum vitamin D levels, group 1 consisted of patients with serum vitamin D levels <20 ng/ml and in group 2 patients with serum vitamin D levels ≥20 ng/ml were included. Pattern of serum ca^2+^, serum PTH and thyroid diseases (malignant vs benign) were studied in these patients. All patients were followed up to 3 months following discharge and any evidence of symptomatic or biochemical hypocalcemia was recorded. Data was collected and was analysed using SPSS statistical software.

## Results

The mean age for patients included in the study was 37.4 ± 15.39 years (range13-65 years) with male to female ratio of 1:6. Out of 35 patients included in our study 19(54.29%) patients underwent hemithyroidectomy and 16 (45.7%) patients had total thyroidectomy (TT). The means ± SD for pre-operative and post-operative serum albumin [n = 35] were 4.79 ± 0.88 g/dl and 4.64 ± 0.82 g/dl respectively. Mean serum vitamin D level of the patients under study was 16.22 ± 12.85 ng/ml. Out of 35 patients, 24 (68.5%) had serum vitamin D level <20 ng/ml and 11(31.5%) had serum vitamin D levels ≥20 ng/ml. In control group of 30 apparently healthy individuals, mean serum vitamin D levels were 20.3 ± 7.7 ng/ml ranging from 9-39 ng/ml. 16 individuals (53.3%) were having serum vitamin D levels ≤20 ng/ml indicating a high prevalence of vitamin D deficiency in our population. Mean pre-op and post-op serum PTH levels [n = 35] were 24.4 ± 10.2 pg/ml and 20 ± 10 pg/ml respectively indicative of a 18% fall in post-operative period which was higher i.e. 35% in patients undergoing TT than in patients undergoing hemithyroidectomy who had a fall of 11%. On applying Pearson Correlation Coefficients there was a significant positive correlation between post-operative serum PTH and postoperative serum ca^2+^ at 24 hrs. (P = 0.0113) but there was no significant correlation between pre-operative vitamin D and pre-operative PTH (p = 0.4155) and post-operative PTH (p = 0.1938).

The difference between pre-op and post-op PTH levels was statistically significant (t = 4.47 and p = 0.001). In patients undergoing TT with or without modified radical neck dissection (MRND) the pre-op and post-op-PTH levels were 23.7 ± 9.2 and 15.3 ± 7.9 pg/ml with a significant difference in pre-operative and post-operative levels (t = 5.08,p = .0001) whereas in patients undergoing hemithyroidectomy mean pre-op and post-op parathormone levels were 25.11 ± 11.1 and 22.5 ± 10.8 (t = 2 p = 0.062), this difference was not statistically significant. Mean pre-op [n = 35] serum ca^2+^ was 9.3 ± 0.54 mg/dl (normally distributed P < 0.037) and mean post-op serum ca^2+^ were 8.7 ± 0.6 mg/dL at 12 hrs (normally distributed P < 0.049), 8.6 ± 0.58 at 24 hrs (not- normally distributed P > 0.15), 8.7 ± 0.43 at 1 week and 8.9 ± 0.31 at 3 months. Difference between mean pre-op serum ca^2+^ and mean post-op serum ca^2+^ at 24 hrs was statistically significant (t = 6.94 and p < 0.001). In patients with serum vitamin D ≤20 ng/ml mean pre-operative and post-operative serum ca^2+^ levels were 9.3 ± 0.5 and 8.4 ± 0.58 g dl (p < .001) whereas in patients with serum vitamin D levels >20 ng/ml mean pre-operative and post-operative serum ca^2+^ were 9.52 ± 0.64 and 8.9 ± 0.5 (p = ns). In patients undergoing TT with or without MRND [n = 16] mean pre-op serum ca^2+^ and mean post-op serum ca^2+^ (at 24 hr) were 9.5 ± 0.4 and 8.34 ± 0.54 with a statistically significant difference. In patients undergoing hemithyroidectomy mean pre-op serum ca^2+^ and post-op serum ca^2+^ (at 24 hrs.) were 9.25 ± 0.6 and 8.82 ± 0.54 with no statistically significant difference. Observations made in two groups i.e. group 1(vitamin D<20ng/ml) and group 2(vitamin D ≥20ng/ml) are summarized in following Table 1.

**Table 1:** **Summary of observations made in two groups i.e. group 1 (vitamin D < 20 ng/ml) and group 2 (vitamin D ≥20 ng/ml)**

Parameters	Serum vitamin D < 20 ng/ml (n = 24)	Serum vitamin D ≥20 ng/ml (n = 11)
Mean pre-operative serum calcium (mg/d)	9.3 ± 0.5	9.52 ± 0.64
Mean post-operative serum calcium (mg/d)	8.4 ± 0.58	8.9 ± 0.5
No. of patients developing hypocalcaemia in post-operative period	11	1
Mean pre-operative serum PTH (pg/ml)	25 ± 11.4	23.2 ± 7.2
Mean post-operative serum PTH (pg/ml)	19.8 ± 11.5	20.4 ± 6.2
% fall in PTH	21%	12%

Out of 24 patients with serum vitamin D levels <20 ng/ml 11(46%) developed post-operative hypocalcemia, it’s worth noting here that overall 12 patients developed post-operative hypocalcemia in the patients under study (n = 35), and 11 (90%) of them were vitamin D deficient. In patients with serum vitamin D levels ≥20 ng/ml only one patient developed post-operative hypocalcemia who had TT.

### Evaluation of patients according to postoperative hypocalcemia

Out of 35 patients included in the study no patient developed symptomatic hypocalcemia in first 12 hrs, however biochemical hypocalcemia was noted in 8 (22.8%) patients. In first 24 hrs 7 patients (20%) developed symptomatic hypocalcemia and 12 (34.3%) patients developed biochemical hypocalcemia. This temporary hypocalcemia disappeared gradually and no patient was symptomatic on 7th POD, however biochemical hypocalcemia persisted in 5 (14%) patients at one week POD. On follow-up after 3 months, no patient was having either symptomatic or biochemical hypocalcemia however 3(8.5%) patients who underwent TT with neck dissection and one (2.8%) who had TT were on oral calcium.

## Discussion

Postoperative hypocalcemia is a major concern following thyroid surgery. When severe, it can lead to serious complications and requires intravenous therapy to alleviate the clinical symptoms. [2] Temporary hypocalcemia if untreated or undiagnosed, may lead to paraesthesia, tetany, and an increased duration of hospital stay with repeated blood tests and increase cost of thyroid surgery [1].

In our study [n = 35], during the first 24 hrs 12 (34.3%) patients developed biochemical hypocalcemia out of which 7 patients (20%) were symptomatic; however 3 (8.5%) patients who underwent TT with neck dissection and one (2.8%) patient who had TT were on oral calcium carbonate on 3 month follow-up indicative of permanent hypoparathyroidism. This data is in concordance with the literature available on this issue [1, 4, 9–12].

### Surgical technique and occurrence of post-operative hypocalcemia

Both Billroth and Kocher noticed occurrence of tetany following thyroid surgery [5]. In last two decades TT has become the procedure of choice for both malignant and benign thyroid diseases leading to a search for ways to reduce the incidence of hypocalcemia following thyroid surgery. One of the most important reasons is the technique of TT (extracapsular vs capsular) and extent of dissection (no nodal dissection vs nodal dissection). In our study, we performed TT only in malignant diseases of thyroid which was often combined with a neck dissection procedure; hence to achieve a better oncological clearance we adopted extracapsular technique. However in TT for benign diseases and in hemithyroidectomy procedure we adhered to capsular dissection. Patients who underwent extracapsular dissection are more likely to develop hypocalcemia as reported by Thomusch et al. [5] They performed bilateral thyroid surgery in 5846 patients for benign and malignant thyroid disease. They concluded that the extent of resection and surgical technique had a greater impact on the rates of permanent postoperative hypoparathyroidism than thyroid pathologic condition. In their study TT, bilateral central ligation of the inferior thyroid artery, identification and preservation of no or only a single parathyroid gland and Graves’ disease emerged as independent risk factors. In bilateral thyroid surgery, even when a standard capsular technique is adopted, results still vary, with apparently inexplicable temporary but not permanent hypocalcemia. Patients undergoing TT without neck dissection 6% developed hypocalcemia in post-operative period however 57% of patient with TT and neck dissection developed hypocalcaemia, more importantly permanent hypocalcemia was more frequent in TT with neck dissection than in TT only (8.5% vs 2.8%). Occurrence of post-operative hypocalcaemia was lesser in patients undergoing TT for benign diseases in whom capsular dissection was applied and in hemithyroidectomy (33% vs 21% respectively). In hemithyroidectomy group (n = 19), 4 patients developed hypocalcemia, all the patients except one developing hypocalcemia in hemithyroidectomy group were having serum vitamin D levels <10 ng/ml with a mean value of 9 ± 5 ng/ml and fall in post-operative serum PTH from its pre-operative level was 30%. In hemithyroidectomy no dissection is done on other half , hence occurrence of hypocalcemia in hemithyroidectomy group is difficult to explain, however one plausible explanation is presence of vitamin D deficiency with severe vitamin D deficiency (<10 ng/ml) in 3 out of 4 patients developing hypocalcemia in post-operative period. In this setting pre-operative vitamin D deficiency appears to be the single most important factor in predicting post-operative hypocalcemia in patients undergoing hemithyroidectomy. In hemithyroidectomy even if one lobe of thyroid is removed there are chances of venous stasis due to oedema in the operative field which temporarily gives rise to a deteriorated vascular supply to the parathyroids [13]. Number of parathyroids vary from 2-6 and there is also a variation in their anatomical location [14]. Hence even if one lobe is handled chances of parathyroid injury cannot be ruled out.

In our study parathyroid auto transplantation was routinely performed whenever a parathyroid gland ischemia or injury was identified .Despite potentially excellent long-term results, the liberal resection and auto transplantation of the parathyroid glands carries a high risk of temporary but clinically significant hypocalcemia [1]. Thomusch et al emphasized that at least 2 parathyroid glands should be identified and preserved to prevent hypoparathyroidism and they found that no additional benefit was evident by having more than two identified and preserved parathyroid glands [5]. The safety of parathyroid auto transplantation is not absolute. Even after auto transplantation of parathyroid glands the graft is not completely revascularized before 3 weeks [15]. Hence patient may develop hypocalcemia in immediate post-operative period even if parathyroid glands are auto-transplanted which disappears later usually after 6-8 weeks. One cannot exclude the progressively increased function of a supernumerary, rudimentary fifth parathyroid gland at a thymic or mediastinal site. In our study [n = 35] 7 patients (20%) received parathyroid gland auto transplantation in their ipsilateral sternocleidomastoid muscle, two parathyroid glands were identified and implanted in 5 patients whereas in the remaining two patients only one parathyroid gland was implanted. Out of 7 patients who received parathyroid auto transplantation, 4(57%) had post-operative hypocalcaemia and 1 (14.3%) had evidence of hypocalcemia at 3 months.

Hence in our study, although parathyroid auto transplantation had no significant impact on early post-operative hypocalcaemia it has got a significant effect when permanent hypoparathyroidism is considered.

Hemodilution is one factor which may be responsible for changes in serum calcium levels in perioperative period and it also explains their occurrence with other extra cervical operations of the same magnitude and duration as with thyroidectomy [9].

In patients with hyperthyroidism, when normal parathyroid function can be documented, “hungry bone syndrome” appears to be the most probable cause of hypocalcaemia in hyperthyroid patients. The risk of hypocalcaemia is not alleviated by the correction of hyperthyroidism within a few weeks before thyroidectomy. It is correlated with the pretreatment serum levels of free thyroxine and with markers of bone turnover rate, such as serum alkaline phosphatase levels and urinary hydroxyproline levels [8, 9].

### Vitamin D deficiency and it’s correlation with the occurrence of post-operative hypocalcaemia

It is now recognized and documented that vitamin D deficiency is one of the most common medical condition in the world [15]. Western data shows a prevalence of 30-50% in both children and adults [16, 17]. Contrary to popular belief that India being a tropical country must have a low prevalence of vitamin D deficiency, several Indian studies have now shown a high prevalence of vitamin D deficiency [18–20]. Vitamin D has a critical role in calcium homeostasis. Like other hormones, 1, 25 (OH)_2_ D (vitamin D3) circulates at picogram concentrations that are 1000 times less than those of the precursor 25(OH)D. Based on the need for increased ca^2+^ absorption, the synthesis of 1,25(OH)_2_D is tightly regulated and stimulated primarily by serum parathyroid hormone (PTH), as well as low serum calcium or phosphorus levels, and inhibited by circulating FGF23 produced by osteocytes. Although produced in the kidney, 1,25(OH)_2_D acts at a distance in the intestinal cell to increase ca^2+^ absorption or in the bone to stimulate differentiation and activation of osteoblasts and osteoclasts [21]. Insufficient ca^2+^ absorption due to low vitamin D concentrations leads to an increase in PTH secretion. Increased PTH stimulates the synthesis of calcitriol and thereby improves ca^2+^ absorption efficiency [22, 23]. We hypothesized that patients with preoperative normal serum vitamin D levels can compensate for postoperative hypocalcemia due to parathyroid injury without clinical and laboratory indications of hypocalcemia. High frequency of vitamin D deficiency could be responsible for the relatively high frequency of temporary postoperative hypocalcemia in our study .We found that low preoperative serum vitamin D were significantly associated with postoperative hypocalcemia. In our study out of 24 patients with serum vitamin D levels <20 ng/ml 11(46%) developed post-operative hypocalcemia, it’s worth noting here that overall 12 patients developed post-operative hypocalcemia in the patients under study (n = 35), and 11 (90%) of them were vitamin D deficient. In patients with serum vitamin D levels ≥20 ng/ml only one patient developed post-operative hypocalcemia who had bilateral surgery in the form of TT. These observations suggest a crucial role of vitamin D deficiency in post-operative hypocalcemia after thyroid surgery.

As already discussed serum calcium levels are regulated by multiple factors, parathormone and vitamin D levels being two most important factors. In patients with parathyroid ischemia or injury, any kind of fall in post-operative serum ca^2+^ levels is unable to increase the secretion of PTH from ischemic/injured parathyroid glands, hence their dependence on serum vitamin D to maintain adequate serum ca^2+^ levels increase. Vitamin D acts on enterocytes to increase absorption of ca^2+^ from intestine. In vitamin D deficient patients this mechanism of increased ca^2+^ absorption is impaired hence these patients fail to maintain their serum ca^2+^ levels in the setting of impaired parathyroid functioning and develop hypocalcemia either biochemical or symptomatic. Similar studies investigating role of serum vitamin D in the occurrence of post-operative hypocalcemia after thyroidectomy has shown concordant results with our study [21, 24, 25].

## Conclusion and recommendation

Pre-operative serum vitamin D levels have got positive correlation with serum calcium levels in early post-operative period. Patients with serum vitamin D levels <20 ng/ml are highly likely to develop early post-operative hypocalcemia and the difference between pre-operative and post-operative serum calcium levels in vitamin D deficient patients was significant (p < 0.001). Occurrence of post-operative symptomatic hypocalcemia is more common in patients with vitamin D deficiency. In our study pre-operative vitamin D deficiency appears to be the single most important factor in predicting post-operative hypocalcemia in patients undergoing unilateral surgery (hemithyroidectomy) in whom parathyroid ischemia and injury is unlikely. We recommend prophylactic administration of vitamin D to prevent occurrence of hypocalcemia in early post-operative period in patients undergoing thyroid surgery.

## Consent

Written informed consent was obtained from all the patients for the publication of this report and any accompanying images.
